# Interleukin-1 Receptor Antagonist and Interleukin-4 Genes Variable Number Tandem Repeats Are Associated with Adiposity in Malaysian Subjects

**DOI:** 10.1155/2017/4104137

**Published:** 2017-02-15

**Authors:** Yung-Yean Kok, Hing-Huat Ong, Yee-How Say

**Affiliations:** Department of Biomedical Science, Faculty of Science, Universiti Tunku Abdul Rahman (UTAR), Kampar Campus, Jalan Universiti, Bandar Barat, 31900 Kampar, Perak, Malaysia

## Abstract

Interleukin-1 receptor antagonist (*IL1RA*) intron 2 86 bp repeat and interleukin-4 (*IL4*) intron 3 70 bp repeat are variable number tandem repeats (VNTRs) that have been associated with various diseases, but their role in obesity is elusive. The objective of this study was to investigate the association of* IL1RA* and* IL4* VNTRs with obesity and adiposity in 315 Malaysian subjects (128 M/187 F; 23 Malays/251 ethnic Chinese/41 ethnic Indians). The allelic distributions of* IL1RA* and* IL4* were significantly different among ethnicities, and the alleles were associated with total body fat (TBF) classes. Individuals with* IL1RA* I/II genotype or allele II had greater risk of having higher overall adiposity, relative to those having the I/I genotype or I allele, respectively, even after controlling for ethnicity [Odds Ratio (OR) of I/II genotype = 12.21 (CI = 2.54, 58.79; *p* = 0.002); II allele = 5.78 (CI = 1.73, 19.29; *p* = 0.004)]. However,* IL4* VNTR B2 allele was only significantly associated with overall adiposity status before adjusting for ethnicity [OR = 1.53 (CI = 1.04, 2.23; *p* = 0.03)]. Individuals with* IL1RA* II allele had significantly higher TBF than those with I allele (31.79 ± 2.52* versus *23.51 ± 0.40; *p* = 0.005). Taken together,* IL1RA* intron 2 VNTR seems to be a genetic marker for overall adiposity status in Malaysian subjects.

## 1. Introduction

The prevalence of obesity worldwide is rising at alarming rate and has been described as a global pandemic. Malaysia has one of the highest rates of obesity in Asia-Pacific, where the combined prevalence of overweight and obesity was 43.8% and 48.6% among men and women above 20 years, respectively [1]. Obesity is closely associated with chronic and low-grade inflammation in the adipose tissue, signified by a lower level of anti-inflammatory cytokines and higher level of proinflammatory cytokines, which in turn differentially activate adipose-tissue macrophages (ATMs) [2]. Specifically, anti-inflammatory cytokines interleukin- (IL-) 13, IL-4, and IL-10 stimulate the alternatively activated ATMs (M2) in lean persons, while obesity induces a shift to the classically activated ATMs (M1) due to stimulation by proinflammatory cytokines TNF-*α*, IL-1*β*, and IL-6 [3].

Interleukin-1 receptor antagonist (IL-1ra), also known as IL-1RN, is an endogenous competitive inhibitor of proinflammatory IL-1*α* and IL-1*β* [4] and is highly secreted by the white adipose tissue (WAT) [5]. IL-1ra is a proadipogenic factor as* IL1RA* knockout mice have reduced adipose storage, impaired adipogenesis, and decrease in adipocyte size [6], while its level is increased in the serum of obese patients, correlating with body mass index (BMI) and insulin resistance [7]. The human IL-1ra gene (*IL1RA *or* IL1RN*) has a Variable Number Tandem Repeat (VNTR) polymorphism within intron 2 due to variation in the number of copies of an 86 bp sequence. To date, six distinct alleles corresponding to 1, 2, 3, 4, 5, and 6 copies of the repeat sequence have been identified [8]. The 4-repeat (allele I) and 2-repeat (allele II) are most frequently found in the general population, while the other four alleles (alleles III, IV, V, and VI) are rarely observed [9]. This VNTR, particularly homozygosity for allele II, has been variably associated with various conditions such as obesity, inflammatory bowel disease, and coronary artery disease [reviewed in [10]] in different ethnic populations worldwide.

IL-4, secreted by activated Th2 lymphocytes, basophils, and mast cells, executes many biological roles such as induction of Th2 differentiation, immunoglobulin class switching, and B cell proliferation [11]. In animal studies, diet-induced obese mice had increased splenic lymphocytes production of IL-4 [12], rats receiving visceral fat removal surgery had decreased serum IL-4 [13], and mice treated with IL-4 had improved insulin sensitivity and glucose tolerance while lipid accumulation in adipose tissues was inhibited [14]. These suggest that IL-4 may participate in the processes of diet-induced obesity and metabolism. Similar to* IL1RA*,* IL4* has a 70 bp VNTR polymorphism within intron 3, and two common alleles are B1 and B2 that have two and three tandem repeats, respectively [15]. There have been several reports on the association between the VNTR B1 allele and inflammatory diseases, such as multiple sclerosis [16], rheumatoid arthritis [17], and systemic lupus erythematosus [18]. With regard to obesity, there are limited studies on this VNTR, where two studies showed no association [19, 20].

Since the association of* IL1RA* and* IL4* VNTRs with obesity and its related parameters is still elusive especially in Asians, the objectives of this study are therefore to investigate the distribution of* IL1RA* and* IL4* VNTRs genotypes and alleles and to determine whether they are associated with overall obesity (as measured by BMI), central adiposity (as measured by waist circumference, WC), and overall adiposity (as measured by total body fat, TBF) in Malaysian subjects.

## 2. Materials and Methods

### 2.1. Subjects

Questionnaire and sample collection were carried out using convenience sampling method among unrelated and nonoverlapping 315 subjects comprising of three cohorts (128 or 40.63% males and 187 or 59.37% females): (1) 69 Universiti Tunku Abdul Rahman (UTAR), Setapak Campus students and residents of Setapak and Petaling Jaya were recruited from October 2009 to February 2010 [32 males and 37 females; 23 Malays, 40 Chinese, and 6 Indians; mean age 28.49 years]; (2) 20 UTAR Perak Campus students were recruited from October 2011 to January 2012 [9 males and 11 females; all Chinese; mean age 19.90 years]; (3) UTAR Perak Campus students were recruited from January 2013 to May 2013 [87 males and 139 females; 192 Chinese and 34 Indians; mean age 21.30 years]. The ethnicities of the subjects were self-identified. All subjects were pooled together for data analysis. This study has received ethical approvals from the UTAR Scientific and Ethical Review Committee (SERC). All subjects signed informed consent forms, and the study was conducted in accordance with the Declaration of Helsinki (amended in Brazil, 2013).

### 2.2. Questionnaire and Anthropometric Measurements

Clinical and anthropometric measurements, namely, systolic blood pressure (SBP), diastolic blood pressure (DBP), pulse rate, waist circumference (WC), hip circumference (HC), waist-to-hip ratio (WHR), weight, height, body mass index (BMI), and total body fat (TBF), were measured as described in our previous studies [21, 22]. The cut-off points for obesity, overall adiposity (TBF), and central adiposity (WHR) were ≥ 25 kg/m^2^ [23], 20% (males) or 30% (females) [24], and 0.90 (males) or 0.85 (females) [25], respectively.

### 2.3. DNA Extraction and Genotyping

**Figure 1:**
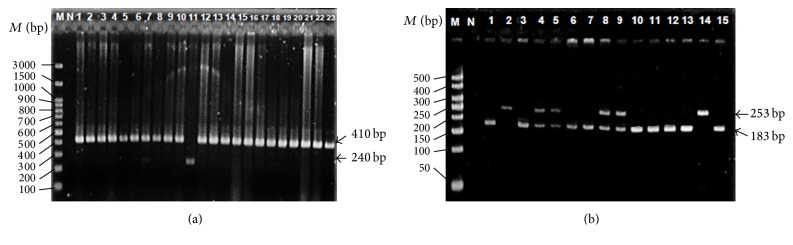
Images of PCR products of (a)* IL1RA* and (b)* IL4 *VNTR in 1.5% or 3.0% agarose gel, respectively. Lane M: 100 bp or 50 bp DNA ladder (Thermo Fischer Scientific, MA, USA). Lane N: Negative Control. Lanes A1–6: 410/410 bp (I/I). Lane A7: 410/240 bp (I/II). Lane A11: 240/240 bp (II/II). Lanes B1 and 3: 183/183 bp (B1/B1). Lanes B2 and 14: 253/253 bp (B2/B2). Lanes B4 and 5: 253/183 bp (B1/B2).

Participants were asked to rinse their vigorously with 5 mL of 3% sucrose solution for 1 min and the mouthwash samples were preserved in 3 mL TNE buffer [17 mM Tris/HCl (pH 8.0), 50 mM NaCl, and 7 mM EDTA]. DNA extraction protocol was then conducted following the protocol as outlined previously [26]. The* IL1RA *and* IL4 *VNTRs were genotyped by Polymerase Chain Reaction using the primers, reagents, and conditions adopted from [27] or [20], respectively, with minor modifications. PCR was performed in a 20 *μ*L total reaction mixture containing 1x PCR buffer (NH_4_)_2_SO_4_ without MgCl_2_ (PhileKorea, Korea), 1.5 mM MgCl_2_, 0.2 mM dNTPs (PhileKorea, Korea), 0.5 *μ*M of forward primer, 0.5 *μ*M of reverse primer, 100 ng of DNA template, 0.5 U of Taq polymerase (PhileKorea, Korea), and an appropriate volume of sterile deionized water to top up. The PCR amplification protocol was carried out using the T100™ Thermal Cycler PCR machine (Bio-Rad, CA, USA) which began with initial denaturation at 95°C for 5 min, followed by 30 cycles of denaturation at 94°C for 30 sec, annealing at 60°C for 30 sec (for* IL1RA*) or 66°C for 45 sec (for* IL4*), extension at 72°C for 30 sec (for* IL1RA*) or 1 min (for* IL4*), and final extension at 72°C for 10 min. PCR products were electrophoresed on either 1.5% (for* IL1RA*) or 3% (for* IL4*) agarose gel, then stained with ethidium bromide, and visualized under UV light after electrophoresis. The sizes of the bands for* IL1RA* alleles were I: 410 bp; II: 240 bp;* IL4* alleles were B2: 253 bp; B1: 183 bp (Figure 1). Three genotypes from each VNTR were verified by DNA sequencing of PCR products (First BASE Laboratories Sdn. Bhd., Malaysia).

### 2.4. Statistical Analysis

Statistical analysis was carried out using the IBM SPSS Statistics software version 16.0 (IBM, NY, USA). Allelic frequencies were estimated by gene counting and the distribution of genotypes was tested for Hardy-Weinberg equilibrium using the Chi-square (*χ*^2^) test. Data for continuous variables were presented as adjusted means ± standard error of the mean (SEM) and as frequency for categorical variables. The normality of distributions of continuous variables was tested with the Kolmogorov-Smirnov test and variables that were not distributed normally were log-transformed prior to statistical analysis. Genotype and allele frequencies of the polymorphism were assessed for association with demographic and anthropometric classes using Pearson's *χ*^2^ test or Fischer's exact test. Logistic regression analysis (enter method) was performed for overall adiposity status with adjustment for covariate ethnicity. Analysis of covariance (ANCOVA) using the univariate General Linear Model with adjustment for covariate ethnicity was performed for anthropometric measurements and blood pressures. ANCOVA was also performed based on BMI status in stratified analysis. A *p* value of less than 0.05 was considered as statistically significant.

## 3. Results

The demographic and anthropometric characteristics of the 315 subjects are as shown in Table 1. There was no significant association between gender and the demographic and anthropometric classes.

Table 2 shows the genotype and allele distribution of* IL1RA *and* IL4 *VNTRs, which did not deviate from the Hardy-Weinberg equilibrium and are categorized under different demographic and anthropometric classes. The overall minor allele frequencies (MAFs) of* IL1RA *and* IL4 *were 0.02 and 0.25, respectively. The allelic distribution of* IL1RA* was significantly different between Chinese and Indians and for* IL4*, it was significantly different between all ethnicities (Table 2).* IL1RA *and* IL4 *allele distributions were also significantly associated with TBF class, but not other demographic and anthropometric classes. Genotype distribution of* IL4* was also not significantly associated with gender, ethnicity, BMI, WC, and TBF classes.

Since TBF class was significantly associated with both* IL1RA* and* IL4* VNTRs, logistic regression analysis was carried out to study the association of* IL1RA* and* IL4* VNTRs with overall adiposity (TBF) status (Table 3). As TBF was significantly different between ethnicities (data not shown), ethnicity was considered as a covariate. The* IL1RA* VNTR genotype and allele were significantly associated with overall adiposity before and after adjusting for ethnicity. Particularly, subjects with heterozygous I/II genotype had 14.45 times higher risk to have high adiposity compared with subjects with homozygous I/I genotype, while those with allele II had 6.81 times higher risk to have high adiposity compared with subjects with allele I. After controlling for ethnicity, the association of* IL1RA* genotype and allele with adiposity status remained significant with I/II subjects having 12.21 times higher risk compared with I/I subjects and II subjects having 5.78 times higher risk compared with I subjects (Table 3). However,* IL4* VNTR B2 allele was only significantly associated with overall adiposity status before adjusting for ethnicity [OR = 1.53 (CI = 1.04, 2.23; *p* = 0.03)], and this association was abolished after controlling for ethnicity [OR = 1.13 (CI = 0.74, 1.74; *p* = 0.57)] (Table 3).

Indeed, covariate analysis of variance after controlling for ethnicity also showed similar result, where subjects carrying* IL1RA* II allele had 8.28% significantly higher TBF than the those with I allele (Table 4). However, stratified analysis based on BMI status showed that this was only true among nonobese subjects, but not obese subjects (data not shown). All other anthropometric measurements and blood pressures were not significantly different between* IL1RA *and* IL4 *VNTR alleles.

## 4. Discussion

In this study, as expected, only allele I and allele II of* IL1RA* VNTR can be found. The other alleles are too rare to be found in most of the populations [27]. The* IL1RA* VNTR is considered a rare genetic polymorphism, as the MAF was 0.02 (less than 0.05). There is significant proof to show that* IL1RA* VNTR is highly influenced by ethnicity [reviewed in [27]]. A study in 19 Chinese populations found out that allele I had higher frequency than allele II, which were 0.913 and 0.064, respectively, indicating that the prevalence of allele I in China was significantly higher, and the prevalence of allele II was significantly lower than American and European Caucasian populations [28]. Similarly in African and African-American people, the frequency of allele II homozygotes is considerably lower than that in the Caucasian population [29]. Alike with* IL1RA*, the allele and genotype frequencies for* IL4* VNTR in our population are strikingly different from those of the Caucasian population [17, 30–32], with our MAF of 0.25 being more similar to the Japanese population (MAF = 0.33) [33] and a previous Malaysian study (MAF = 0.37) [34].

IL-1ra serum level is increased in human obesity and is under strong genetic control [5], partly by the* IL1RA* VNTR polymorphism. Indeed,* IL1RA* allele II has a clear influence on IL-1ra circulating levels since, in normal human subjects, its carrier individuals had higher levels than the noncarrier individuals (745 ng/mL* versus* 627 pg/mL) [35]. With regard to obesity, two previous Asian studies with relatively small sample sizes found no significant association of* IL1RA* VNTR with BMI in Koreans (*N* = 261) [36] and North Indians (*N* = 103) [37]. Similarly, our study found no association with BMI value or overall obesity status, but* IL1RA* VNTR was associated with both TBF value and overall adiposity status. In stratified analysis, TBF value was found to be higher in subjects carrying* IL1RA* II allele than those with I allele, only among nonobese subjects. This could be attributed to the low BMI and high TBF paradox among Malays/Chinese/Indians, as shown previously [38]. The* IL1RA *VNTR may have functional significance as the repeated sequence contains possible binding sites for transcription factors [9]. A review by Witkin et al. 2002 [10] summarized that individuals homozygous for allele II have more prolonged and severe proinflammatory immune response than persons with other* IL1RA* genotypes, which might be beneficial when combating infectious agents [39] or neoplasms [40], but is detrimental for those with chronic inflammatory conditions [41] or those who are pregnant [42].

The* IL4* VNTR which is located in* IL4 *intron 3 could be a functional polymorphism as it could affect mRNA splicing, leading to different splice variants [43]. Indeed, the B2 allele has been associated with reduced amount of peripheral Th cells which produce IL-4 [33]. Our study showed that this VNTR was associated with overall adiposity status (TBF class), but not with TBF value after adjustment for ethnicity. However, consistent with two previous studies which showed no association of this VNTR with obesity status in North Indian [19] and Turkish [20] populations, we found no association with both BMI value and overall obesity status. The role of IL-4 in modulating adipogenesis has been established by previous studies. Tsao et al. (2014) [44] showed IL-4 inhibited adipogenesis via STAT6 pathway or by influencing the cell proliferation at the mitotic clonal expansion phase. IL-4 also enhances lipolysis via the PKA pathway in mature adipocytes [44]. Other study carried out by Rao et al. (2014) [45] revealed that IL-4 signaling activates macrophages in WAT, leading to the production of nervous system molecules required for converting WAT to beige fat. Taken together, the direct functional significance of* IL1RA* and* IL4* VNTRs particularly on affected tissues in obesity (like adipose tissue) and other metabolic disease (like type 2 diabetes mellitus) warrants further investigation.

Limitations of the present study include the small sample size of Malay and Indian subjects and the lack of other indigenous ethnic groups especially from East Malaysia (Sabah and Sarawak); hence the results from this study may not be fully representative of the general Malaysian population. Other common genetic variants like the* IL-1β* promoter region and exon-5 and* IL4* -590 T/C single nucleotide polymorphisms, which were not screened in this study, could have association with obesity and its related parameters. The direct phenotype-genotype correlation could also be determined by measuring the serum levels of IL-1ra and IL-4 using ELISA in the future.

Obesity results in a proinflammatory state which involves the release of cytokines and adipokines by adipose tissue. Indeed, the main finding of this study shows that VNTR genetic polymorphisms in two genes,* IL1RA* and* IL4*, encoding for anti-inflammatory cytokines IL-1ra and IL-4, respectively, are associated with overall adiposity status (TBF) in Malaysian subjects, before controlling for ethnicity.* IL1RA* VNTR shows a more prominent effect, as TBF was significantly higher in those with* IL1RA* II allele compared with I allele, even after controlling for ethnicity. Individuals with* IL4* VNTR B2 allele had higher risk of having higher adiposity, but the association was abolished after controlling for ethnicity. Taken together,* IL1RA* intron 2 VNTR seems to be a genetic marker for overall adiposity status in Malaysian subjects.

## Figures and Tables

**Table 1:** Demographic and anthropometric characteristics of the subjects according to gender.

Variables	Male (*n* = 128)	Female (*n* = 187)
Ethnicity		
Malay	11 (8.6)	12 (6.4)
Chinese	104 (81.2)	147 (78.6)
Indian	13 (10.2)	28 (15.0)
*χ*^2^; *p*	1.91; 0.38	
BMI class		
Nonobese	103 (80.5)	158 (84.5)
Obese	25 (19.5)	29 (15.5)
*χ*^2^; *p*	0.87; 0.35	
WC class		
Normal	104 (81.2)	147 (78.6)
High	24 (18.8)	40 (21.4)
*χ*^2^; *p*	0.33; 0.57	
TBF class		
Normal	84 (65.6)	137 (73.3)
High	44 (34.4)	50 (26.7)
*χ*^2^; *p*	2.12; 0.15	

Parentheses indicate percentage (%) within the same gender.

**Table 2:** Association of *IL1RA *and* IL4* VNTRs genotype and allele distribution with demographic and anthropometric classes.

Genotypes/alleles	Gender		Ethnicity			BMI class		WC class		TBF class	
	Male	Female	Malay	Chinese	Indian	Nonobese	Obese	Normal	High	Normal	High
*IL1RA*											
I/I	124 (96.9)	177 (94.7)	23 (100)	245 (97.6)	33 (80.5)	250 (95.8)	51 (94.4)	241 (96.0)	60 (93.8)	218 (98.6)	83 (88.3)
I/II	4 (3.1)	9 (4.8)	0	5 (2.0)	8 (19.5)	10 (3.8)	3 (5.6)	9 (3.6)	4 (6.2)	2 (0.9)	11 (11.7)
II/II	0	1 (0.5)	0	1 (0.4)	0	1 (0.4)	0	1 (0.4)	0	1 (0.5)	0
*χ*^2^; *p*	NP										
I	252 (98.4)	363 (97.1)	46 (100)	495 (98.6)	74 (90.2)	510 (97.7)	105 (97.2)	491 (97.8)	124 (96.9)	438 (99.1)	177 (94.1)
II	4 (1.6)	11 (2.9)	0	7 (1.4)	8 (9.8)	12 (2.3)	3 (2.8)	11 (2.2)	4 (3.1)	4 (0.9)	11 (5.9)
*p*	0.30		M *versus* C: 1.00; M *versus* I: 0.05; C *versus* I: <0.001^*∗*^			0.73		0.52		0.001^*∗*^	
*IL4*											
B1/B1	84 (65.6)	108 (57.8)	10 (43.5)	167 (66.5)	15 (36.6)	160 (61.3)	32 (59.3)	153 (61.0)	39 (60.9)	141 (63.8)	51 (54.3)
B1/B2	31 (24.2)	57 (30.5)	11 (47.8)	67 (26.7)	10 (24.4)	74 (28.4)	14 (25.9)	69 (27.5)	19 (29.7)	60 (27.1)	28 (29.8)
B2/B2	13 (10.2)	22 (11.8)	2 (8.7)	17 (6.8)	16 (39.0)	27 (10.3)	8 914.8)	29 (11.6)	6 (9.4)	20 (9.0)	15 (16.0)
*χ*^2^; *p*	2.02; 0.37		NP			0.93; 0.63		0.31; 0.86		3.98; 0.14	
B1	199 (77.7)	273 (73.0)	31 (67.4)	401 (79.9)	40 (48.8)	394 (75.5)	78 (72.2)	375 (74.7)	97 (75.8)	342 (77.4)	130 (69.1)
B2	57 (22.3)	101 (27.0)	15 (32.6)	101 (20.1)	42 (51.2)	128 (24.5)	30 (27.8)	127 (25.3)	31 (24.2)	100 (22.6)	58 (30.9)
*χ*^2^; *p*	1.82; 0.18		37.78; <0.001^*∗*^			0.51; 0.48		0.06; 0.80		4.75; 0.03^*∗*^	

Parentheses indicate percentage within the same demographic/anthropometric class; NP = *χ*^2^ test not performed due presence of cell having the count of less than 5; *p* values by *χ*^2^ or Fisher's exact test; ^*∗*^*p* value significant at <0.05; *p* values for ethnicity are combination analysis of Malay *versus* Chinese, Malay *versus* Indian, and Chinese *versus* Indian.

**Table 3:** Logistic regression analysis for the association of *IL1RA* and *IL4* VNTR with overall adiposity status.

Genotype/allele	Unadjusted		Adjusted^§^	
	Odds ratio (95% CI)	*p*	Odds ratio (95% CI)	*p*
*IL1RA *(*n*; %)				
I/I (301; 95.6)	1.00		1.00	
I/II (13; 4.1)	14.45 (3.14, 66.56)	0.001^*∗*^	12.21 (2.54, 58.79)	0.002^*∗*^
II/II (1; 0.3)	NP	1.00	NP	1.00
I (615; 97.6)	1.00		1.00	
II (15; 2.4)	6.81 (2.14, 21.66)	0.001^*∗*^	5.78 (1.73, 19.29)	0.004^*∗*^
*IL4 *(*n*; %)				
B1/B1 (192; 61.0)	1.00		1.00	
B1/B2 (88; 27.9)	2.07 (0.99, 4.36)	0.05	1.32 (0.57, 3.06)	0.53
B2/B2 (35; 11.1)	1.29 (0.74, 2.24)	0.37	1.01 (0.55, 1.85)	0.97
B1 (472; 74.9)	1.00		1.00	
B2 (158; 25.1)	1.53 (1.04, 2.23)	0.03^*∗*^	1.13 (0.74, 1.74)	0.57

^§^Adjusted for covariate ethnicity; values are by logistic regression enter method; ^*∗*^*p* value significant at <0.05.

**Table 4:** Adjusted means of overall population's anthropometric measurements and blood pressures for different *IL1RA *and *IL4 *VNTR alleles.

Variables	*IL1RA*		*IL4*	
	I	II	B1	B2
WC	77.16 ± 0.48	81.98 ± 3.06	79.59 ± 1.59	79.55 ± 2.64
*p*	0.09		0.99	
BMI	21.83 ± 0.18	23.26 ± 1.12	22.54 ± 0.58	22.55 ± 0.97
*p*	0.14		0.95	
TBF	23.51 ± 0.40	31.79 ± 2.52	26.09 ± 1.31	29.21 ± 2.18
*p*	0.005^*∗*^		0.34	
SBP	113.26 ± 0.68	108.95 ± 4.32	114.65 ± 2.24	107.56 ± 3.73
*p*	0.19		0.06	
DBP	68.81 ± 0.41	68.66 ± 2.60	68.22 ± 1.35	69.25 ± 2.25
*p*	0.68		0.37	

WHR: waist-to-hip ratio; BMI: body mass index; TBF: total body fat; SBP: systolic blood pressure; DBP: diastolic blood pressure; all values were log-transformed before analysis by univariate analysis of variance (General Linear Model), adjusted for covariate ethnicity; values are presented as adjusted mean ± SEM (estimated marginal means ± standard error of the mean); ^*∗*^*p* valuesignificant at < 0.05.
